# Bis(2,3-dimethyl­anilinium) tetra­chlorido­zincate dihydrate

**DOI:** 10.1107/S1600536811017478

**Published:** 2011-05-14

**Authors:** Sofiane Souissi, Wajda Smirani Sta, Salem S. Al-Deyab, Mohamed Rzaigui

**Affiliations:** a Laboratoire de Chimie des Matériaux, Faculté des Sciences de Bizerte, 7021 Zarzouna Bizerte, Tunisia; b Petrochemical Research Chair, College of Science, King Saud University, Riyadh, Saudi Arabia

## Abstract

In the title compound, (C_8_H_12_N)_2_[ZnCl_4_]·2H_2_O, the Zn atom is coordinated by four Cl atoms in a tetra­hedral geometry. The water mol­ecules and the organic cations inter­act with the [ZnCl_4_]^2−^ complex anions, building up a two-dimensional hydrogen-bonded network parallel to (100).

## Related literature

For properties of aniline derivatives, see: Hirao & Fukuhara (1998 ▶); Linden *et al.* (1995 ▶); MacDiamid *et al.* (1998 ▶); Singh *et al.* (1995 ▶, 2002 ▶); Wang *et al.* (2002 ▶); Fábry *et al.* (2002 ▶). For structural comparison, see: Harrison (2005 ▶); Marouani *et al.* (2010 ▶).
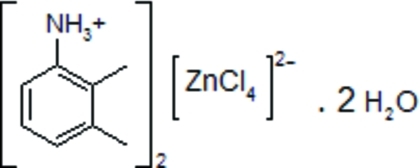


## Experimental

### 

#### Crystal data

(C_8_H_12_N)_2_[ZnCl_4_]·2H_2_O
*M* *_r_* = 487.57
Monoclinic, 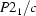

*a* = 21.654 (2) Å
*b* = 7.432 (3) Å
*c* = 14.069 (2) Å
β = 90.30 (2)°
*V* = 2264.1 (10) Å^3^
*Z* = 4
Ag *K*α radiation
λ = 0.56085 Å
μ = 0.82 mm^−1^
*T* = 293 K
0.35 × 0.30 × 0.25 mm

#### Data collection

Enraf–Nonius TurboCAD-4 diffractometer
16232 measured reflections
10928 independent reflections
5697 reflections with *I* > 2σ(*I*)
*R*_int_ = 0.041
2 standard reflections every 120 min  intensity decay: 5%

#### Refinement

*R*[*F* ^2^ > 2σ(*F* ^2^)] = 0.054
*wR*(*F* ^2^) = 0.151
*S* = 1.03
10363 reflections
232 parameters
H-atom parameters constrained
Δρ_max_ = 0.77 e Å^−3^
Δρ_min_ = −0.92 e Å^−3^

### 

Data collection: *CAD-4 EXPRESS* (Enraf–Nonius, 1994 ▶); cell refinement: *CAD-4 EXPRESS*; data reduction: *XCAD4* (Harms & Wocadlo, 1996 ▶); program(s) used to solve structure: *SHELXS97* (Sheldrick, 2008 ▶); program(s) used to refine structure: *SHELXL97* (Sheldrick, 2008 ▶); molecular graphics: *ORTEPIII* (Burnett & Johnson, 1996 ▶), *ORTEP-3 for Windows* (Farrugia, 1997 ▶) and *PLATON* (Spek, 2009 ▶); software used to prepare material for publication: *WinGX* (Farrugia, 1999 ▶).

## Supplementary Material

Crystal structure: contains datablocks I, global. DOI: 10.1107/S1600536811017478/dn2685sup1.cif

Structure factors: contains datablocks I. DOI: 10.1107/S1600536811017478/dn2685Isup2.hkl

Additional supplementary materials:  crystallographic information; 3D view; checkCIF report

## Figures and Tables

**Table 1:** Hydrogen-bond geometry (Å, °)

*D*—H⋯*A*	*D*—H	H⋯*A*	*D*⋯*A*	*D*—H⋯*A*
N1—H1*A*⋯Cl2^i^	0.89	2.61	3.488 (2)	168
N1—H1*B*⋯Cl4	0.89	2.38	3.239 (2)	162
N1—H1*C*⋯O1	0.89	1.83	2.707 (3)	168
N2—H2*A*⋯Cl2^ii^	0.89	2.85	3.713 (2)	165
N2—H2*B*⋯Cl3	0.89	2.35	3.225 (2)	168
N2—H2*C*⋯O2	0.89	1.82	2.696 (3)	167
O1—H22⋯Cl1^iii^	0.80	2.35	3.115 (2)	160
O1—H23⋯Cl4^i^	0.81	2.53	3.304 (3)	162
O2—H20⋯Cl3^i^	0.80	2.56	3.228 (3)	142
O2—H21⋯Cl1	0.79	2.50	3.213 (2)	150

Symmetry codes: (i) 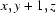; (ii) 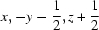; (iii) 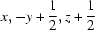.

**Fig. 1.**
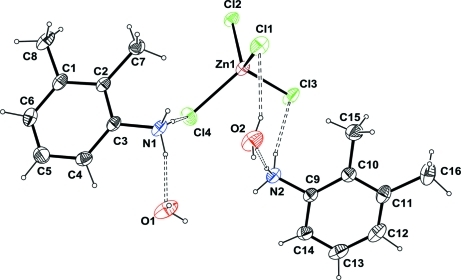
The assymetric unit of the title compound, with the atom numbering scheme. Displacement ellipsoids are drawn at the 30% probability level. H atoms are represented as small sphere of arbitrary radii. Hydrogen bonds are shown as dashed lines.

**Fig. 2.**
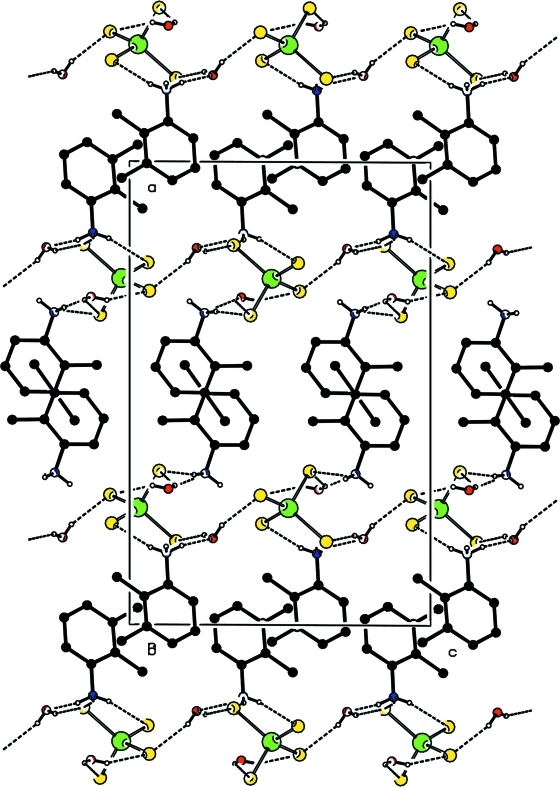
A view of the atomic arrangement of the title compound along the b axis.

## supplementary crystallographic information

### Comment

Aniline is an useful chemical product used in various areas. Some derivatives of aniline have improving anticorrosion ability for metals (Wang *et al.*, 2002), others show high efficiency as chemical sensors (MacDiamid *et al.*,1998) and catalitic oxidation (Hirao & Fukuhara, 1998). Bibliography reports some structures where the cation dimethylanilinium is associated to other anions as sulfate (Singh *et al.*, 2002), nitrate, perchlorate (Singh *et al.*, 1995), chloride (Linden *et al.*, 1995), and phosphate (Fábry *et al.*, 2002). We report here a crystal structure where this organic cation is associated to an anionic complex (I).

The asymmetric unit consists of two 2,3-dimethylanilinium cations, two water molecules and one complex anion [ZnCl_4_]^2- ^linked by N-H···O, N-H···Cl and O-H···Cl hydrogen bonds (Fig. 1). The atomic arrangement of (2,3-(CH_3_)_2_C_6_H_3_NH_3_)_2_ZnCl_4_.2H_2_O (I) is made up of inorganic layers, parallel to the (1 0 0) plane, built up by [ZnCl_4_]^2-^ complex and water molecules held together by O—H···Cl hydrogen bonds. The organic groups are attached to both sides of these layers through N—H···Cl and N—H···O hydrogen bonds, electrostatic and Van der walls interactions, to form a two dimensional infinite network (Fig. 2).

In the title compound (I), the four chlorine atoms of the [ZnCl_4_]^2-^ anion are acting as acceptors of the hydrogen bonds. The bond angles Cl—Zn—Cl vary from 102.50 (3) to 113.71 (3)°, and the bond length of the Zn—Cl lie in the range 2.2071 (8) - 2.4649 (9) Å. These values indicate that the coordination geometry of the Zn atom can be considered as being a slightly distorted tetrahedron (Harrison, 2005). The nearst Zn···Zn intra-chain separation is 7.135 (1) Å, while the distance between adjacent chains is 11.050 (2) Å. The examination of the organic cations shows that the value distances and angles show no significant difference from those obtained in other crystals involving the same organic groups (Marouani *et al.*, 2010). The phenyl rings of these cations are planar with a maximum atomic deviation of 0.00025 Å and a dihedral angle between them of 21.95°.

### Experimental

A mixture of an aqueous solution of 2,3-xylidine, HCl and ZnCl_2_ in a 2:2:1 molar ratio was prepared, stirred then slowly evaporated at room temperature (293 K). After few days, colourless prismatic crystals of (C_16_H_28_N_2_) [ZnCl_4_].H_2_O appear with suitable size for *x*-ray diffraction measurements.

### Refinement

All H atoms attached to C atoms and N atom were fixed geometrically and treated as riding with C—H = 0.96 Å (methyl) or 0.93 Å (aromatic) and N—H = 0.89 Å with U_iso_(H) = 1.2U_eq_(C_aromatic_) or U_iso_(H) = 1.5U_eq_(C_methyl_,N). H atoms of water molecule were located in difference Fourier maps and included in the subsequent refinement using restraints (O-H= 0.82 (1)Å and H···H= 1.37 (2)Å) with U_iso_(H) = 1.5U_eq_(O). In the last cycle of refinement, they were treated as riding on their parent O atoms.

### Crystal data

**Table:**

(C_8_H_12_N)_2_[ZnCl_4_]·2H_2_O	*F*(000) = 1008
*M**_r_* = 487.57	*D*_x_ = 1.430 Mg m^−^^3^
Monoclinic, *P*2_1_/*c*	Ag *K*α radiation, λ = 0.56085 Å
Hall symbol: -P 2ybc	Cell parameters from 25 reflections
*a* = 21.654 (2) Å	θ = 9–11°
*b* = 7.432 (3) Å	µ = 0.82 mm^−^^1^
*c* = 14.069 (2) Å	*T* = 293 K
β = 90.30 (2)°	Block, colourless
*V* = 2264.1 (10) Å^3^	0.35 × 0.30 × 0.25 mm
*Z* = 4	

### Data collection

**Table:**

Enraf–Nonius TurboCAD-4 diffractometer	*R*_int_ = 0.041
Radiation source: fine-focus sealed tube	θ_max_ = 28.0°, θ_min_ = 2.3°
graphite	*h* = −36→2
non–profiled ω scans	*k* = −3→12
16232 measured reflections	*l* = −23→23
10928 independent reflections	2 standard reflections every 120 min
5697 reflections with *I* > 2σ(*I*)	intensity decay: 5%

### Refinement

**Table:**

Refinement on *F*^2^	Primary atom site location: structure-invariant direct methods
Least-squares matrix: full	Secondary atom site location: difference Fourier map
*R*[*F*^2^ > 2σ(*F*^2^)] = 0.054	Hydrogen site location: inferred from neighbouring sites
*wR*(*F*^2^) = 0.151	H-atom parameters constrained
*S* = 1.03	*w* = 1/[σ^2^(*F*_o_^2^) + (0.0738*P*)^2^] where *P* = (*F*_o_^2^ + 2*F*_c_^2^)/3
10363 reflections	(Δ/σ)_max_ = 0.005
232 parameters	Δρ_max_ = 0.77 e Å^−^^3^
0 restraints	Δρ_min_ = −0.92 e Å^−^^3^

### Special details

**Table:**

Geometry. All esds (except the esd in the dihedral angle between two l.s. planes) are estimated using the full covariance matrix. The cell esds are taken into account individually in the estimation of esds in distances, angles and torsion angles; correlations between esds in cell parameters are only used when they are defined by crystal symmetry. An approximate (isotropic) treatment of cell esds is used for estimating esds involving l.s. planes.
Refinement. Refinement of *F*^2^ against ALL reflections. The weighted *R*-factor *wR* and goodness of fit *S* are based on *F*^2^, conventional *R*-factors *R* are based on *F*, with *F* set to zero for negative *F*^2^. The threshold expression of *F*^2^ > σ(*F*^2^) is used only for calculating *R*-factors(gt) *etc*. and is not relevant to the choice of reflections for refinement. *R*-factors based on *F*^2^ are statistically about twice as large as those based on *F*, and *R*- factors based on ALL data will be even larger.

### Fractional atomic coordinates and isotropic or equivalent isotropic displacement parameters (Å^2^)

**Table:**

	*x*	*y*	*z*	*U*_iso_*/*U*_eq_	
Zn1	0.252371 (12)	−0.16993 (3)	0.528190 (18)	0.03788 (8)	
Cl1	0.27972 (3)	0.07344 (10)	0.43474 (5)	0.05745 (18)	
Cl2	0.21828 (3)	−0.39507 (9)	0.44011 (4)	0.04583 (14)	
Cl3	0.33196 (3)	−0.28623 (10)	0.59834 (5)	0.05693 (18)	
Cl4	0.18023 (3)	−0.07803 (10)	0.65412 (5)	0.05405 (16)	
N1	0.15472 (9)	0.3472 (3)	0.62226 (15)	0.0432 (5)	
H1A	0.1666	0.4037	0.5697	0.065*	
H1B	0.1682	0.2341	0.6210	0.065*	
H1C	0.1704	0.4032	0.6728	0.065*	
C1	−0.00434 (11)	0.2889 (3)	0.55691 (16)	0.0390 (5)	
C2	0.06097 (10)	0.2868 (3)	0.54830 (14)	0.0337 (4)	
C3	0.08621 (10)	0.3481 (3)	0.62776 (15)	0.0352 (4)	
C4	0.05039 (12)	0.4082 (4)	0.71143 (15)	0.0433 (5)	
H4	0.0725	0.4497	0.7638	0.052*	
C5	−0.01386 (13)	0.4076 (4)	0.71794 (18)	0.0496 (6)	
H5	−0.0352	0.4463	0.7713	0.060*	
C6	−0.04063 (11)	0.3476 (3)	0.64175 (18)	0.0455 (5)	
H6	−0.0835	0.3408	0.6397	0.055*	
C7	0.10203 (12)	0.2261 (4)	0.45764 (17)	0.0469 (5)	
H7A	0.1423	0.1895	0.4790	0.070*	
H7B	0.1059	0.3256	0.4145	0.070*	
H7C	0.0822	0.1275	0.4258	0.070*	
C8	−0.03606 (13)	0.2301 (4)	0.47333 (19)	0.0528 (7)	
H8A	−0.0230	0.1104	0.4575	0.079*	
H8B	−0.0268	0.3099	0.4217	0.079*	
H8C	−0.0797	0.2304	0.4846	0.079*	
N2	0.32690 (9)	0.0370 (3)	0.75104 (14)	0.0449 (5)	
H2A	0.3019	0.0255	0.8007	0.067*	
H2B	0.3236	−0.0597	0.7141	0.067*	
H2C	0.3164	0.1345	0.7180	0.067*	
C9	0.38814 (10)	0.0540 (3)	0.78301 (15)	0.0370 (4)	
C10	0.44164 (11)	0.0658 (3)	0.71609 (16)	0.0392 (5)	
C11	0.49906 (12)	0.0739 (3)	0.75038 (19)	0.0476 (6)	
C12	0.49854 (14)	0.0754 (4)	0.8488 (2)	0.0633 (8)	
H12	0.5369	0.0839	0.8783	0.076*	
C13	0.44415 (15)	0.0651 (4)	0.9136 (2)	0.0618 (8)	
H13	0.4507	0.0672	0.9789	0.074*	
C14	0.38810 (12)	0.0534 (4)	0.88115 (16)	0.0458 (5)	
H14	0.3529	0.0457	0.9186	0.055*	
C15	0.44037 (14)	0.0705 (5)	0.60951 (17)	0.0584 (7)	
H15A	0.4667	0.1654	0.5874	0.088*	
H15B	0.3989	0.0917	0.5879	0.088*	
H15C	0.4547	−0.0426	0.5851	0.088*	
C16	0.56043 (13)	0.0778 (5)	0.6831 (3)	0.0686 (9)	
H16A	0.5623	−0.0309	0.6463	0.103*	
H16B	0.5967	0.0871	0.7223	0.103*	
H16C	0.5583	0.1795	0.6411	0.103*	
O1	0.18655 (11)	0.5435 (3)	0.77698 (15)	0.0708 (6)	
H22	0.2147	0.4997	0.8063	0.106*	
H23	0.1938	0.6349	0.7481	0.106*	
O2	0.29296 (11)	0.2985 (3)	0.62872 (17)	0.0694 (6)	
H20	0.3001	0.3971	0.6497	0.104*	
H21	0.3001	0.2703	0.5757	0.104*	

### Atomic displacement parameters (Å^2^)

**Table:**

	*U*^11^	*U*^22^	*U*^33^	*U*^12^	*U*^13^	*U*^23^
Zn1	0.03496 (13)	0.03231 (13)	0.04624 (15)	−0.00035 (10)	−0.01116 (10)	−0.00039 (11)
Cl1	0.0599 (4)	0.0473 (4)	0.0651 (4)	−0.0146 (3)	−0.0099 (3)	0.0139 (3)
Cl2	0.0435 (3)	0.0433 (3)	0.0506 (3)	−0.0065 (2)	−0.0105 (2)	−0.0068 (2)
Cl3	0.0492 (3)	0.0477 (3)	0.0736 (4)	0.0098 (3)	−0.0305 (3)	−0.0102 (3)
Cl4	0.0570 (4)	0.0474 (4)	0.0578 (3)	0.0109 (3)	0.0097 (3)	0.0049 (3)
N1	0.0379 (9)	0.0407 (11)	0.0508 (10)	−0.0003 (8)	−0.0184 (8)	0.0025 (9)
C1	0.0378 (10)	0.0280 (9)	0.0511 (12)	−0.0022 (9)	−0.0153 (9)	0.0082 (9)
C2	0.0355 (10)	0.0258 (9)	0.0397 (10)	−0.0004 (8)	−0.0097 (8)	0.0028 (8)
C3	0.0343 (10)	0.0295 (10)	0.0416 (10)	0.0011 (8)	−0.0112 (8)	0.0040 (8)
C4	0.0494 (13)	0.0440 (13)	0.0363 (10)	0.0034 (11)	−0.0096 (9)	0.0023 (10)
C5	0.0523 (14)	0.0528 (16)	0.0439 (12)	0.0089 (12)	0.0023 (11)	0.0071 (11)
C6	0.0345 (11)	0.0441 (13)	0.0579 (14)	0.0004 (10)	−0.0052 (10)	0.0122 (11)
C7	0.0455 (12)	0.0456 (13)	0.0494 (12)	0.0001 (11)	−0.0075 (10)	−0.0090 (11)
C8	0.0521 (14)	0.0380 (12)	0.0680 (16)	−0.0076 (11)	−0.0301 (12)	0.0042 (12)
N2	0.0385 (10)	0.0503 (12)	0.0458 (10)	−0.0011 (9)	−0.0087 (8)	0.0019 (9)
C9	0.0386 (10)	0.0295 (10)	0.0427 (11)	0.0009 (9)	−0.0088 (9)	−0.0016 (9)
C10	0.0419 (11)	0.0317 (10)	0.0439 (11)	−0.0016 (9)	−0.0035 (9)	−0.0002 (9)
C11	0.0390 (11)	0.0336 (11)	0.0702 (16)	−0.0032 (10)	−0.0105 (11)	0.0016 (11)
C12	0.0550 (16)	0.0554 (17)	0.0791 (19)	−0.0041 (14)	−0.0303 (15)	−0.0023 (15)
C13	0.0702 (19)	0.0612 (18)	0.0537 (15)	−0.0002 (16)	−0.0254 (14)	−0.0045 (14)
C14	0.0497 (13)	0.0462 (14)	0.0414 (11)	0.0022 (11)	−0.0065 (10)	−0.0017 (10)
C15	0.0564 (16)	0.073 (2)	0.0455 (13)	−0.0108 (15)	−0.0007 (12)	0.0042 (13)
C16	0.0426 (14)	0.0590 (19)	0.104 (3)	−0.0093 (14)	−0.0012 (15)	0.0018 (18)
O1	0.0872 (16)	0.0537 (12)	0.0712 (12)	−0.0128 (12)	−0.0381 (11)	−0.0002 (10)
O2	0.0762 (15)	0.0501 (12)	0.0815 (14)	0.0020 (11)	−0.0265 (12)	0.0059 (10)

### Geometric parameters (Å, °)

**Table:**

Zn1—Cl3	2.1618 (7)	N2—C9	1.404 (3)
Zn1—Cl2	2.2069 (8)	N2—H2A	0.8900
Zn1—Cl1	2.3149 (9)	N2—H2B	0.8900
Zn1—Cl4	2.4648 (8)	N2—H2C	0.8900
N1—C3	1.486 (3)	C9—C14	1.381 (3)
N1—H1A	0.8900	C9—C10	1.499 (3)
N1—H1B	0.8900	C10—C11	1.333 (3)
N1—H1C	0.8900	C10—C15	1.500 (3)
C1—C2	1.420 (3)	C11—C12	1.384 (4)
C1—C8	1.427 (3)	C11—C16	1.636 (4)
C1—C6	1.498 (4)	C12—C13	1.495 (5)
C2—C3	1.323 (3)	C12—H12	0.9300
C2—C7	1.623 (3)	C13—C14	1.297 (4)
C3—C4	1.482 (3)	C13—H13	0.9300
C4—C5	1.395 (4)	C14—H14	0.9300
C4—H4	0.9300	C15—H15A	0.9600
C5—C6	1.295 (4)	C15—H15B	0.9600
C5—H5	0.9300	C15—H15C	0.9600
C6—H6	0.9300	C16—H16A	0.9600
C7—H7A	0.9600	C16—H16B	0.9600
C7—H7B	0.9600	C16—H16C	0.9600
C7—H7C	0.9600	O1—H22	0.8041
C8—H8A	0.9600	O1—H23	0.8069
C8—H8B	0.9600	O2—H20	0.8042
C8—H8C	0.9600	O2—H21	0.7908
			
Cl3—Zn1—Cl2	102.52 (3)	H8A—C8—H8C	109.5
Cl3—Zn1—Cl1	111.47 (3)	H8B—C8—H8C	109.5
Cl2—Zn1—Cl1	111.06 (3)	C9—N2—H2A	109.5
Cl3—Zn1—Cl4	106.82 (3)	C9—N2—H2B	109.5
Cl2—Zn1—Cl4	113.72 (3)	H2A—N2—H2B	109.5
Cl1—Zn1—Cl4	110.90 (3)	C9—N2—H2C	109.5
C3—N1—H1A	109.5	H2A—N2—H2C	109.5
C3—N1—H1B	109.5	H2B—N2—H2C	109.5
H1A—N1—H1B	109.5	C14—C9—N2	108.3 (2)
C3—N1—H1C	109.5	C14—C9—C10	129.3 (2)
H1A—N1—H1C	109.5	N2—C9—C10	122.40 (19)
H1B—N1—H1C	109.5	C11—C10—C9	119.9 (2)
C2—C1—C8	113.7 (2)	C11—C10—C15	111.9 (2)
C2—C1—C6	126.7 (2)	C9—C10—C15	128.2 (2)
C8—C1—C6	119.6 (2)	C10—C11—C12	110.5 (3)
C3—C2—C1	109.4 (2)	C10—C11—C16	123.4 (2)
C3—C2—C7	122.3 (2)	C12—C11—C16	126.1 (2)
C1—C2—C7	128.32 (18)	C11—C12—C13	128.3 (2)
C2—C3—C4	124.0 (2)	C11—C12—H12	115.8
C2—C3—N1	111.3 (2)	C13—C12—H12	115.8
C4—C3—N1	124.71 (19)	C14—C13—C12	121.8 (2)
C5—C4—C3	125.3 (2)	C14—C13—H13	119.1
C5—C4—H4	117.4	C12—C13—H13	119.1
C3—C4—H4	117.4	C13—C14—C9	110.2 (3)
C6—C5—C4	112.9 (3)	C13—C14—H14	124.9
C6—C5—H5	123.6	C9—C14—H14	124.9
C4—C5—H5	123.6	C10—C15—H15A	109.5
C5—C6—C1	121.7 (2)	C10—C15—H15B	109.5
C5—C6—H6	119.1	H15A—C15—H15B	109.5
C1—C6—H6	119.1	C10—C15—H15C	109.5
C2—C7—H7A	109.5	H15A—C15—H15C	109.5
C2—C7—H7B	109.5	H15B—C15—H15C	109.5
H7A—C7—H7B	109.5	C11—C16—H16A	109.5
C2—C7—H7C	109.5	C11—C16—H16B	109.5
H7A—C7—H7C	109.5	H16A—C16—H16B	109.5
H7B—C7—H7C	109.5	C11—C16—H16C	109.5
C1—C8—H8A	109.5	H16A—C16—H16C	109.5
C1—C8—H8B	109.5	H16B—C16—H16C	109.5
H8A—C8—H8B	109.5	H22—O1—H23	116.7
C1—C8—H8C	109.5	H20—O2—H21	123.3

### Hydrogen-bond geometry (Å, °)

**Table:**

*D*—H···*A*	*D*—H	H···*A*	*D*···*A*	*D*—H···*A*
N1—H1A···Cl2^i^	0.89	2.61	3.488 (2)	168
N1—H1B···Cl4	0.89	2.38	3.239 (2)	162
N1—H1C···O1	0.89	1.83	2.707 (3)	168
N2—H2A···Cl2^ii^	0.89	2.85	3.713 (2)	165
N2—H2B···Cl3	0.89	2.35	3.225 (2)	168
N2—H2C···O2	0.89	1.82	2.696 (3)	167
O1—H22···Cl1^iii^	0.80	2.35	3.115 (2)	160
O1—H23···Cl4^i^	0.81	2.53	3.304 (3)	162
O2—H20···Cl3^i^	0.80	2.56	3.228 (3)	142
O2—H21···Cl1	0.79	2.50	3.213 (2)	150

Symmetry codes: (i) *x*, *y*+1, *z*; (ii) *x*, −*y*−1/2, *z*+1/2; (iii) *x*, −*y*+1/2, *z*+1/2.
